# Characteristics of Pork Muscles Cooked to Varying End-Point Temperatures

**DOI:** 10.3390/foods10122963

**Published:** 2021-12-02

**Authors:** Reagan N. Cauble, Jase J. Ball, Virginia E. Zorn, Tristan M. Reyes, Madison P. Wagoner, Madison M. Coursen, Barry D. Lambert, Jason K. Apple, Jason T. Sawyer

**Affiliations:** 1 Department of Animal Science, University of Arkansas, Fayetteville, AR 72701, USA; rncauble@uark.edu (R.N.C.); jase.ball@zoetis.com (J.J.B.); 2 Department of Animal Sciences, Auburn University, Auburn, AL 36849, USA; vez0001@auburn.edu (V.E.Z.); tzr0039@auburn.edu (T.M.R.); mpw0035@auburn.edu (M.P.W.); mmc0067@auburn.edu (M.M.C.); 3 Department of Animal Sciences, Tarleton State University, Stephenville, TX 76402, USA; blambert@tarleton.edu; 4 Department of Animal Sciences and Veterinary Technology, Texas A&M University—Kingsville, Kingsville, TX 78363, USA; jason.apple@tamuk.edu

**Keywords:** cooking loss, instrumental cooked color, pork, Warner–Bratzler shear force

## Abstract

M. biceps femoris (BF), m. semimembranosus (SM) and m. semitendinosus (ST) from fresh pork ham were evaluated for characteristics of quality after cooking to an internal endpoint temperature of 62 °C or 73 °C. Fresh ham muscles from the left side (*N* = 68) were cut into 2.54 cm thick chops and allocated to cooking loss, Warner–Bratzler shear force (WBSF), pH and instrumental cooked color analysis. Cooking losses were greater (*p* < 0.0001) for SM and chops cooked to an internal temperature of 73 °C (*p* < 0.0001), whereas WBSF did not differ (*p* = 0.2509) among the three muscles, but was greater (*p* < 0.0001) in chops cooked to 73 °C. Fresh muscle’s pH was greater (*p* < 0.05) in ST than BF or SM. Lastly, the interactive effect (*p* < 0.05) of muscle × endpoint temperature for ST chops cooked to 73 °C was lighter (L*), but, when cooked to 62 °C, they were more red (a*), more yellow (b*) and incurred less color change from red to brown than BF or SM. The current results suggest it is plausible for BF, SM and ST to be considered for alternative uses instead of traditional value-added manufacturing.

## 1. Introduction

Meat and meat products comprise a large percentage of the human diet [1]. Global meat consumption has continually risen from 23.10 kg per person per year in 1961, to 42.20 kg in 2011 [2]. The increased human consumption of meat has resulted in a correlated increase in final market hog live weight from 109.32 kg in 1962 to 128.82 kg in 2014, according to previous [3] market analytics. Additionally, a growing demand for leaner pork products has modified pork production practices [4] through genetic improvements for feed conversion, lean tissue development and reduction of the time to market weight [4].

In response to consumer demand for a healthier, leaner product, the pork value program initiated by the National Pork Producer’s Council (NPPC) in the 1980s, has led to a steady change in carcass merit buying programs resulting in a pork product with decreased adipose tissue and increased muscle development [5]. In accordance with the merit buying program, the United States has moved toward genetic selection for increased lean growth in pork carcasses; however, poor-quality meat characteristics have been associated with this variation in production practices for pigs. Altered production practices have reportedly affected post mortem pH decline, post rigor calpastatin activity and overall tenderness [6]. In addition, as previous authors have noted, changes in meat quality of pork resulting in greater carcass lean weight can cause declines in palatability and acceptability of pork products among consumers [7].

Pork eating quality is affected by a combination of appearance, flavor, tenderness and juiciness [8]. To meet these consumer-focused expectations, the pork industry has established pork quality standards so that pork muscles presented in a retail setting are firm, without exudation and possess a pinkish–red color [9]. Nonetheless, the industry relies heavily on fresh color as a quality indicator and a method to identify substandard pork in pork carcasses and retail cuts. Conditions affecting the structural integrity of post mortem muscle can affect overall meat quality and functionality [5] and may influence carcass merchandising values, along with cooked or processed quality [10]. 

Fresh pork ham is typically used in highly processed products such as sausages and value-added formulations. Fresh pork ham is rarely considered for use in a retail cut setting. However, some examples of value-added ham processing include but are not limited to water-added hams, smoked, cured, or spiral-cut hams. Further processed pork products generally require a large percentage of fresh pork ham, which can be smoked or roasted using a dry or liquid curing brine that alters the final product’s color, flavor and tenderness [11].

Today’s health-conscious consumer now perceives pork as a healthy, tasty, low fat food staple at the time of meat purchasing in the retail setting [5]. Therefore, an analysis muscles from the fresh pork ham could provide another fresh pork option in a retail setting and possibly increase pork carcass’ value. Thus, a greater understanding of the factors affecting fresh and cooked pork muscle’s characteristics is necessary. Therefore, the objective of this study is to identify the cooked characteristics of the biceps femoris (BF), semimembranosus (SM) and semitendinosus (ST) muscles from pork ham.

## 2. Materials and Methods

### 2.1. Raw Materials 

Cross-bred gilts were harvested using humane slaughter conditions under USDA (EST. no. M85B, JBS Swift and Co., Beardstown, IL, USA) inspection at a commercial processing facility. Carcasses were blast-chilled after standard harvesting procedures for 90 min, then fabricated into primals. The left ham from pork carcasses (*N* = 68) was identified, vacuum packaged and shipped under refrigerated conditions to the University of Arkansas Abattoir (Fayetteville, AR, USA) for further fabrication and dissection into subprimals. Hams were skinned using a hand-held pork skinner (S-1011, Best & Donovan, Cincinnati, OH, USA), fabricated into BF, SM and ST and trimmed practically free of subcutaneous fat. Whole muscles were identified based upon the corresponding ham identification number from the processing facility. Muscles with identical packing numbers were vacuum-packaged together and stored at 0 °C for 24 h. 

After individually identifying and vacuum-packaging in barrier bags (3mil, Koch Supplies, North Kansas City, MO, USA), the muscles were transported under refrigerated conditions to the Tarleton State University meat laboratory (Stephenville, TX, USA), where they were stored at −10 °C for 72 h. Subprimals were placed into a blast freezer at −20 °C for 45 min for crust freezing prior to cutting. Chops were cut from the frozen muscles using a commercial band saw (44SFH-LP, Biro, Marblehead, OH, USA) set to 2.54 cm; then, chops were vacuum-packaged individually using a double chamber vacuum-packaging machine (C500, Multivac Inc., Kansas City, MO, USA) and labeled with individual identification. The total number of chops collected per muscle from each ham was quantified with a collection of six chops from BF and SM and four chops from ST from each subprimal (*N* = 68) per muscle. After cutting, packaging and labeling, all chops were stored at −10 °C until laboratory procedures could be completed. 

Chops were analyzed for objective tenderness, moisture loss, pH and instrumental cooked color. Chops were assigned randomly to an end-point internal cooking temperature of either 62 °C or 73 °C. Three chops from each BF and SM were randomly assigned to tenderness and cooking loss, the remaining three chops were analyzed for pH and instrumental cooked color. Four chops were collected from the ST and randomly assigned. Two chops were assigned to measure tenderness and cooking loss and the remaining two chops were analyzed for pH and instrumental cooked color. 

### 2.2. Cooking Loss

Chops were thawed using refrigerated conditions for 24 h at 2 °C and were then removed from packaging prior to cooking. Chops were identified using Styrofoam trays (2s, Walton’s Inc., Wichita, KS, USA) with corresponding muscle type, identification number and internal end-point cooking temperature. Thawed chops were removed from their individual packages, patted dry with a paper towel to remove excess moisture and weighed. A digital scale (ML1501E, Mettler Toledo, LLC, Columbus, OH, USA) was calibrated and an empty Styrofoam tray was placed onto the scale and zeroed. After all raw weights were recorded, chops were placed on a griddle (Model 07061, National Presto Industries Inc., Eau Claire, WI, USA) pre-heated to 176.6 °C. During cooking, chops were flipped every two minutes to insure even cooking occurred. The internal temperature of each chop was taken using a handheld digital thermometer (Model C28 K, Koch Supplies, North Kansas City, MO, USA) after six minutes of cooking. Once the geometric center of the chop reached the required cooking temperature, chops were removed from the griddle, placed onto a tray and allowed to cool to room temperature (23 °C) according to recommended procedures outlined by [12]. 

Cooled chops were weighed after cooking and the cooked weight of each chop was recorded. Cooking loss was calculated by subtracting the cooked weight from the initial raw weight; that number was then divided by the raw weight and multiplied by 100, as follows: [Raw Weight − Cooked Weight] ÷ Raw Weight × 100. Only after collecting cooking loss data was Warner–Bratzler shear force testing conducted on the same chops. 

### 2.3. Warner–Bratzler Shear Force 

The chops used for measuring WBSF were cooked using the same procedures as described above for measuring cooking loss. Upon completion of cooking, cooling of chops to room temperature (23 °C) and cooking loss analysis, no less than six, 1.27 cm diameter cores were taken parallel to the muscle fiber orientation from each chop using a manual cork borer. Each core was sheared once in the center with a Warner–Bratzler compression V-notch cutting blade attachment on an Instron Universal Testing Machine (Instron Corp., Canton, MA, USA), equipped with a 490 N load cell and a crosshead speed of 250 mm/min [12]. The peak forces were averaged for the six cores and used to represent the shear force value of the chop. Kilograms of shear force were converted to Newtons of force (kg of shear force × 9.8). 

### 2.4. Fresh Muscle pH

The chops selected for pH analysis were thawed under refrigerated conditions for 24 h at 2 °C prior to measuring fresh/thawed muscle pH. Intramuscular pH readings were taken using a glass pH probe (IM2100, Pelican Products, South Deerfield, MA, USA) which was calibrated before testing using a 4 pH and a 7 pH (TW-2500-4 and TW-2500-7, ThermoWorks, American Fork, UT, USA) buffer. Three readings were taken by inserting the probe into three random locations within each chop and recorded. The three readings were averaged to determine the fresh muscle pH per chop.

### 2.5. Instrumental Cooked Color

The chops allocated to cooked color measurements were cooked using the same procedures as described previously for cooking loss and WBSF. After cooking and cooling at room temperature (23 °C) for three minutes prior to slicing, instrumental color lightness (L*), redness (a*) and yellowness (b*) were measured. Prior to collecting instrumental color values, the colorimeter was calibrated using standard black and white tiles [12]. The chops were cut just off the center (parallel to the chop flat surface) and color readings were taken immediately across the exposed surface. Instrumental color readings were measured using a Hunter MiniScan EZ (Model 4500 L, HunterLab, Reston, VA, USA). The L*, a* and b* values were determined from the mean of three readings on the surface of each chop using illuminant A, a 10° standard observer and a 25 mm viewing aperture. Lastly, reflectance values within the spectral range from 400 to 700 nm were used to capture the surface color changes from red to brown by calculating the reflectance ratio of 630 nm:580 nm [13]. 

### 2.6. Experimental Design and Statistical Analysis 

The data collected throughout this study were analyzed using the PROC Mixed procedures of SAS (SAS Institute, Inc. Cary, NC). All data were analyzed as a 3 × 2 factorial design with three muscles (BF, SM and ST) and two end-point internal cooking temperatures (62 and 73 °C). Muscle, end-point internal cooking temperature and their interaction were fixed effects whereas chop served as the random effect. The analysis of variance was generated using the mixed models procedure, for moisture retention (cooking loss), instrumental tenderness (WBSF), pH and instrumental internal cooked color. Least squares means were generated using the Kenward–Roger fixed effect SE method and, when significant (*p* < 0.05) F-values were observed, least squares means were separated using pair-wise t-tests. 

## 3. Results and Discussion

### 3.1. Cooking Loss

**Figure 1:**
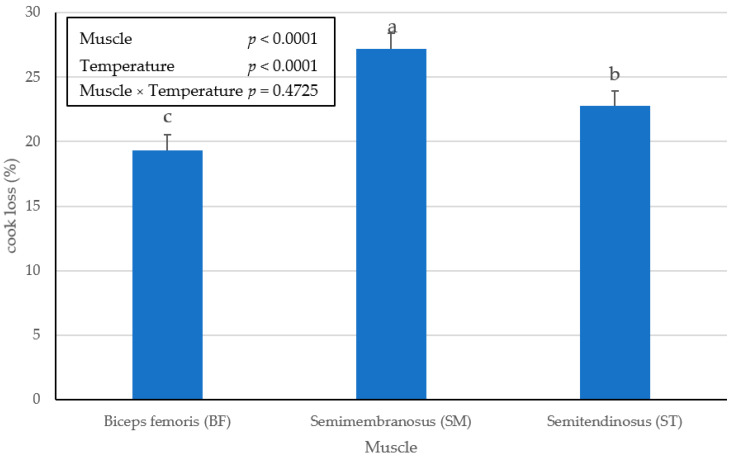
Main effect of ham muscle on cooking loss (%) in pork ham muscles (BF, SM and ST) cooked to an internal temperature of 62 °C or 73 °C. Bars lacking a common superscript differ (*p* ≤ 0.05).

**Figure 2:**
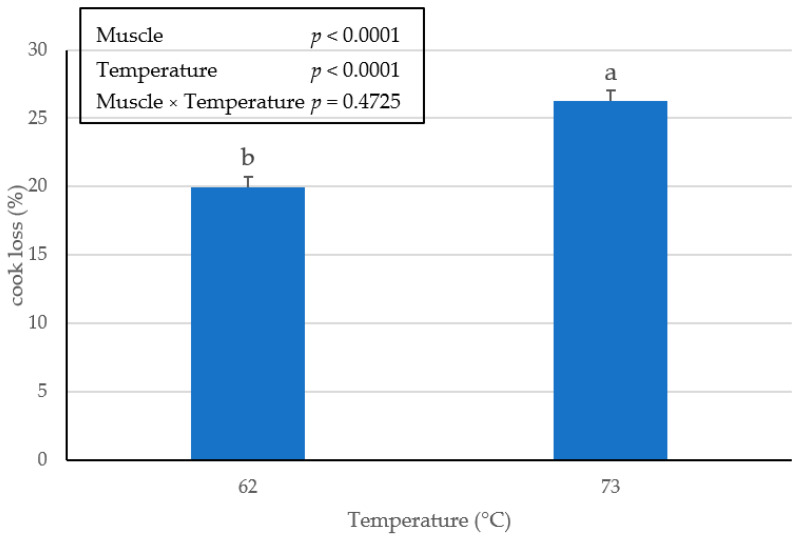
Main effect of endpoint temperature on cooking loss (%) in pork ham muscles (BF, SM and ST) cooked to an internal temperature of 62 °C or 73 °C. Bars lacking a common superscript differ (*p* ≤ 0.05).

There was no interactive effect (*p* > 0.4725) of muscle × temperature on cooking loss of the BF, SM, or ST ham chops. However, there was a significant (*p* < 0.0001) main effect of the muscle as SM expelled the greatest percentage of water during the cooking process (Figure 1) and BF displaced the least. Furthermore, a significant (*p* < 0.0001) main effect of endpoint temperature was detected for pork ham chops cooked (Figure 2) to a 73 °C internal cooking temperature, resulted in a greater percentage of cooking loss than chops cooked to 62 °C. These results agree with previous findings [14], when comparing pork loin chops cooked to endpoint temperatures of 60 °C or 80 °C. 

In another study, boneless loin chops were evaluated for cooked characteristics when cooked to an internal temperature of 63 °C [15]. It was reported that cooking loss percentage across 284 loin chops averaged 16.46% [15]. In comparison, ham chops in the current study cooked to a 62 °C endpoint temperature averaged 27% cooking loss, suggesting that BF, SM and ST could result in variation within the sensory spectrum of tenderness and juiciness because of greater cooking loss. 

In an additional pork muscle study [16], where eight different pork roasts were cooked to four internal temperatures, it was reported that, as the cooking temperature increased, a subsequent increase in cooking loss occurred. Furthermore, these authors indicated that the boneless ham roast had the greatest cooking loss, which could be in relation to the variation in quality of ham muscles. 

Few, if any, previous studies have evaluated the individual muscles from pork ham. However, similar research work [17] evaluating beef steaks from the biceps femoris, longissimus lumborum and the deep pectoralis, cooked to nine endpoint temperatures and using various cooking methods, reported a greater cooking loss for the biceps femoris than the longissimus or pectoralis steaks, regardless of endpoint temperature or cookery method. Even though the previous findings for cooking loss occurred in beef muscles from the beef round, the results of meat cookery in pork ham cuts concur that these muscles can be considered dryer and tougher cuts. 

### 3.2. Warner-Bratlzer Shear Force

**Figure 3:**
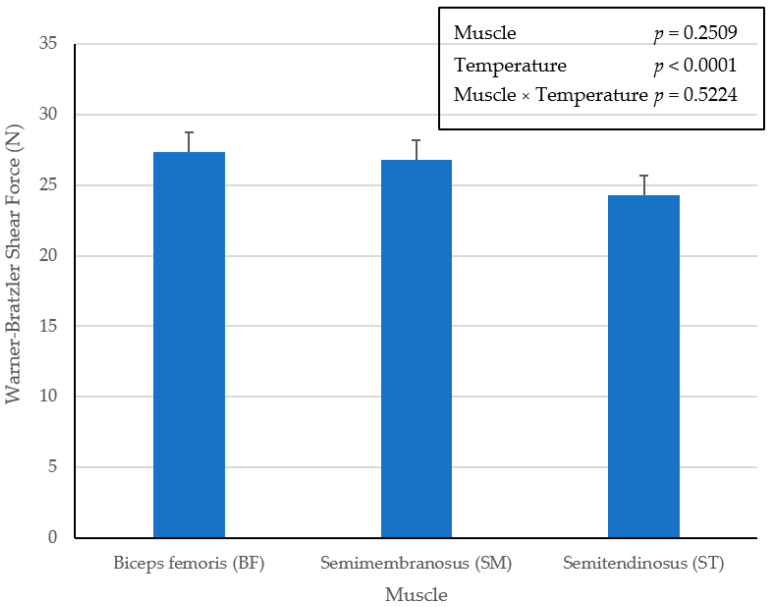
Main effect of ham muscle on Warner–Bratzler shear force (N) in pork ham muscles (BF, SM and ST) cooked to an internal temperature of 62 °C or 73 °C. Bars lacking a common superscript differ (*p* ≤ 0.05).

**Figure 4:**
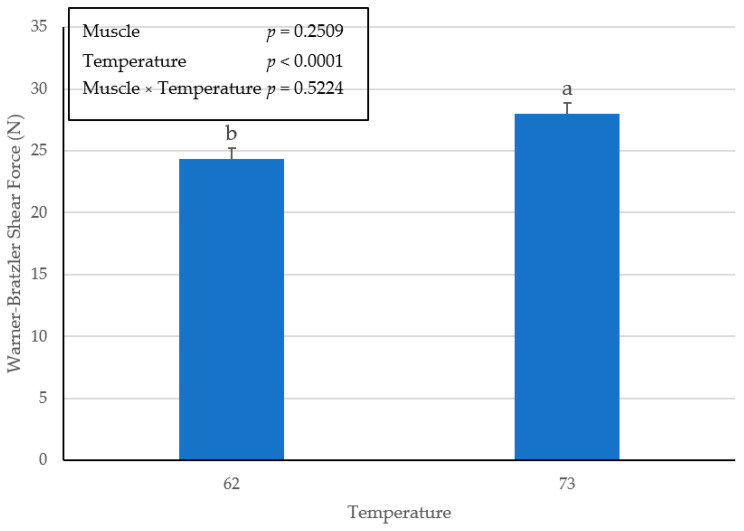
Main effect of endpoint temperature on Warner–Bratzler shear force in pork ham muscles (BF, SM and ST) cooked to an internal temperature of 62 °C or 73 °C. Bars lacking a common superscript differ (*p* ≤ 0.05).

Regardless of the lack of interactive (*p* = 0.5524) effect of muscle × temperature, there were no differences (*p* = 0.2509) in WBSF for the main effect of muscles from pork ham (Figure 3). However, the main effect for WBSF of ham chops cooked to an internal temperature of 73 °C were significantly greater (*p* < 0.0001) than ham chops cooked to an internal endpoint temperature of 62 °C (Figure 4). Unfortunately, sensory taste panels were not conducted during this study to support additional assessments of tenderness evaluation for these muscles from pork ham.

A Warner–Bratzler shear force analysis conducted previously [14] reported that increased end-point cooking temperatures can result in an increase in shear force values. Furthermore, it was noted [14] that instrumental tenderness values for myofibrillar force increased as endpoint temperature increased, but the same instrumental measures for connective tissue force have been reported to decrease. 

In contrast, additional results [16] did not report a significant correlation between increased endpoint temperature and shear force. These variations reported for instrumental tenderness values for pork muscles may be attributed to modifications in experimental design, cookery method, muscle, or cutting technique (roast vs. chop) for retail marketing. 

Interestingly, it has been reported [17] that the beef biceps femoris is more tender when cooked between 40 °C and 60 °C and tenderness decreased when the endpoint cooking temperature increased to 80 °C. Changes in tenderness as result of cooking are likely due to a greater amount of collagen in the biceps femoris and less collagen solubilization at a greater degree of doneness. Surprisingly, when compared to the longissimus lumborum, the biceps femoris was tougher for both cooking methods, regardless of endpoint temperature and cookery method [17]. 

### 3.3. Fresh Muscle pH

**Figure 5:**
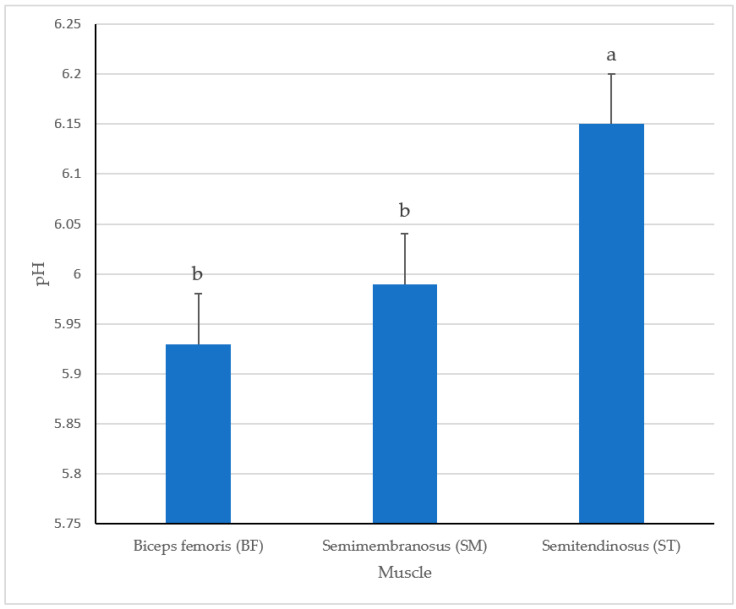
Fresh muscle pH of pork ham muscles (BF, SM and ST). Bars lacking a common superscript differ (*p* ≤ 0.05).

Muscle pH (Figure 5) of fresh ham chops was captured prior to conducting cookery analysis. Values of fresh muscle pH were greater (*p* < 0.0002) in ST than the either BF or SM. The current values for pH concur with previous research results [18] reporting in longissimus dorsi chops cooked to three internal temperatures with greater pH values reported to have a redder (a*) internal color. It is possible that the influence on internal cooked color may be attributed to the ability of a higher pH inhibiting myoglobin from thermal denaturation [19]. Furthermore, it has been reported [18] that b* values could also be affected by a greater muscle pH value. The results for fresh muscle pH of the current study within ST agree with the previous studies that report greater pH values and subsequent greater yellowness (b*) values. 

In addition to internal color, cooking losses recorded in the current study agree with previous results [8] reporting that lower pH values can increase, leading to greater cooking loss. Moreover, this same study [8] reported that, as pH values increased, little to no difference in cooking loss occurred. As noted previously, SM recorded the greatest cooking loss which could be attributed to the lower pH value. 

### 3.4. Instrumental Cooked Color

There was an interactive effect (*p* < 0.05) for muscle and end-point temperature on all instrumental cooked color values (Table 1). The SM was lighter (*p* ≤ 0.0338) when cooked to an internal temperature of 73 °C than all other muscles, regardless of end-point temperature. In addition, internal cooked redness was greater (*p* < 0.0001) for the BF and ST ham chops cooked to an end-point temperature of 62 °C. However, ham chops from ST were more yellow (*p* < 0.0001) than either BF or SM, regardless of internal temperature. Changes from red to brown of ham chops during the cooking process were greatest (*p* < 0.0001) in SM when cooked to an internal temperature of 73 °C. 

The internal cooked color results tend to agree with a previous research work [18] reporting that increased endpoint temperatures decreased the redness (a*) values for longissimus dorsi chops. It is widely known that the current results for internal cooked color are attributed to myoglobin denaturation that occurs in fresh meat during the cooking process. Moreover, these results support that increasing internal temperature of pork ham chops results in decreased myoglobin, causing lower redness (a*) values [19,20]. 

Previous research works have suggested [13,16] that pork should be cooked to an internal temperature of 71.1 °C. The results from this study tend to agree that a degree of doneness of 73 °C may be considered more ideal than 62 °C due to consumer preferences and assumptions on cooked color for pork meat products. Even though chops cooked to 62 °C were redder than those cooked to 73 °C, historically, an internal appearance that is browner/grayer for pork products has been perceived to be safer by consumers.

## 4. Conclusions

The current results suggest that fresh muscles cut into chops from BF, SM and ST and cooked to a cooler degree of doneness result in a more objectively tender product. It is apparent that consumer or trained sensory panel evaluation would have further supported these initial findings on pork ham quality. However, these initial findings lend support that muscles from pork ham may have a plausible role in the retail counter for consumer fresh meat purchases. It should be noted, additional research evaluating underutilized pork muscles from ham and shoulder may provide access to new consumer-friendly retail portions. A focus on simulated retail display, lipid oxidation and instrumental fresh color would enhance the realm of knowledge for these pork cuts in a fresh retail application. 

## Figures and Tables

**Table 1:** Interactive effect of temperature × muscle for instrumental internal cooked color of muscles from pork ham.

	**Endpoint Temperature**								
	62 °C				73 °C				
	**BF**	**SM**	**ST**		**BF**	**SM**	**ST**	SEM *	***p*-value**
Lightness (L*) ^1^	73.79 ^c^	75.85 ^b^	70.76 ^d^		73.77 ^c^	77.41 ^a^	73.05 ^c^	0.527	0.0338
Redness (a*) ^2^	14.61 ^b^	13.45 ^c^	19.75 ^a^		13.41 ^c^	12.14 ^d^	12.92 ^cd^	0.329	0.0001
Yellowness (b*) ^3^	15.24 ^c^	15.43 ^bc^	21.01 ^a^		15.76 ^b^	15.40 ^bc^	15.79 ^b^	0.191	0.0001
From Red to Brown (630:580) ^4^	1.86 ^b^	1.73 ^c^	2.41 ^a^		1.51 ^de^	1.46 ^e^	1.56 ^d^	0.037	0.0001

^1^ Lightness (L*) values are a measure from darkness to lightness (larger value indicates a lighter color; 100 is white and 0 is black); ^2^ Redness (a*) values are a measure of redness (larger value indicates a redder color; +60 is red and −60 is green); ^3^ Yellowness (b*) values are a measure of yellowness (larger value indicates a more yellow color; +60 is yellow and −60 is blue); ^4^ Red:Brown is a calculated ratio of spectral values 630 nm:580 nm which represents a change in color from red to brown (larger value indicates a redder color); ^a,b,c,d,e^ Means lacking a common superscript letter differ (*p* ≤ 0.05); * SEM, standard error of the mean of the interaction.

## References

[1] Risvik E. (1994). Sensory properties and preferences. Meat Sci..

[2] Sans P., Combris P. (2015). World meat consumption patterns: An overview of the last fifty years (1961–2011). Meat Sci..

[3] USDA National Agricultural Statistical Service. 2014. Livestock Slaughter. http://usda.mannlib.cornell.edu/MannUsda/viewDocumentInfo.do?documentID=1101.

[4] Diamant R., Watts B., Cliplef R. (1976). Consumer Criteria for Pork Related to Sensory, Physical and Descriptive Attributes1. Can. Inst. Food Sci. Technol. J..

[5] National Pork Producers Council (2000). Pork Composition and Quality Assessment Procedures.

[6] Huff-Lonergan E., Kuhlers D.L., Lonergan S.M., Mikel W.B., Jungst S.B., Dale S., Reed V.D. (1997). Pork quality decline in response to selection for lean growth efficiency in the absence of the halothane gene. J. Anim. Sci..

[7] Cameron N. (1990). Genetic and phenotypic parameters for carcass traits, meat and eating quality traits in pigs. Livest. Prod. Sci..

[8] Aaslyng M.D., Bejerholm C., Ertbjerg P., Bertram H.C., Andersen H.J. (2003). Cooking loss and juiciness of pork in relation to raw meat quality and cooking procedure. Food Qual. Prefer..

[9] Wachholz D., Kauffman R.G., Henderson D., Lochner J.V. (1978). Consumer discrimination of pork color at the market place. J. Food Sci..

[10] Adorni G., Bianchi D., Cagnoni S. Ham quality control by means of fuzzy decision trees: A case study. Proceedings of the IEEE International Conference on Fuzzy Systems Proceedings.

[11] Mendoza F., Valous N.A., Allen P., Kenny T.A., Ward P., Sun D.-W. (2009). Analysis and classification of commercial ham slice images using directional fractal dimension features. Meat Sci..

[12] American Meat Science Association (AMSA) (2015). Research Guidelines for Cookery, Sensory Evaluation, and Instrumental Tenderness Measurments of Meat.

[13] American Meat Science Association (AMSA) (2012). Meat Color Measurement Guidelines.

[14] Crawford S., Moeller S., Zerby H., Irvin K., Kuber P., Velleman S., Leeds T. (2010). Effects of cooked temperature on pork tenderness and relationships among muscle physiology and pork quality traits in loins from Landrace and Berkshire swine. Meat Sci..

[15] Wilson K.B., Overholt M.F., Shull C.M., Schwab C., Dilger A.C., Boler D.D. (2017). The effects of instrumental color and extractable lipid content on sensory characteristics of pork loin chops cooked to a medium-rare degree of doneness1. J. Anim. Sci..

[16] Heymann H., Hedrick H., Karrasch M., Eggeman M., Ellersieck M. (1990). Sensory and Chemical Characteristics of Fresh Pork Roasts Cooked to Different Endpoint Temperatures. J. Food Sci..

[17] Obuz E., Dikeman M., Grobbel J., Stephens J., Loughin T. (2004). Beef longissimus lumborum, biceps femoris, and deep pectoralis Warner–Bratzler shear force is affected differently by endpoint temperature, cooking method, and USDA quality grade. Meat Sci..

[18] Mancini R., Kropf D., Hunt M., Johnson D. (2005). Effects of endpoint temperature, ph, and storage time on cooked internal color reversion of pork longissimus chops. J. Muscle Foods.

[19] Trout G.R. (1989). Variation in Myoglobin Denaturation and Color of Cooked Beef, Pork, and Turkey Meat as Influenced by pH, Sodium Chloride, Sodium Tripolyphosphate, and Cooking Temperature. J. Food Sci..

[20] Bertram H.C., Engelsen S.B., Busk H., Karlsson A.H., Andersen H.J. (2004). Water properties during cooking of pork studied by low-field NMR relaxation: Effects of curing and the RN^−^-gene. Meat Sci..

